# Supplementary material for: Isolation and Functional Characterization of a Constitutive Promoter in Upland Cotton (Gossypium hirsutum L.)
Source: Int J Mol Sci. 2024 Feb 5;25(3):1917. doi: 10.3390/ijms25031917 (PMC10855717; doi:10.3390/ijms25031917)
Supplement: Supplementary file 1 [file ijms-25-01917-s001.zip › ijms-2783236-supplementary.pdf]

Supplementary Materials

Table S1. pGhFDH nucleic acid sequence.

Promoter Name	Sequence
pGhFDH	GAAAATCCACAACACCTTGCTCGGCTGTCATTTTCGAGAAAAAAATATCTTA<br>ATTCGAAATTTATTGGATAGTTTAAGTTTTGTGGCATATACATTTTAACCAA<br>AGAAAAAAAACCATGCAGTGGGTTATTAATGGAAATCCAACCGATTGCCTG<br>AATCTGAACCATACCCTGAACTTGAAATCGGGCAGGATTTCCTGTTCCACAA<br>ACCCAACATGTTGAACCAAAGAATCCAGAACCTTCTCTATTCGGTGTGCAA<br>GTGGTGCTTTGTGGTTTAGACCTTTAAGTCTGCTGCTTTACTACTACCTATTT<br>ATTTCAAGTAAAGATGGTGGTTATGTATTTTTATAGATATGTGCAACTAAGG<br>CTTAAACAGCTTATGCCAGCTGCTACCTTCAAAAGGAGAACCTCCCAACTAC<br>TCCCTCCAATGCAATCTTAATTATGGGTTTATTTGTCTTTGCACATTATTTGA<br>GTGCAAAAACAAAATGGAAGGCAAAGGATAAGACTGAATATCCAAAAAAA<br>AATTCACAAGAAAATGTAGGTTCCACAAACAACTTTATATCACACATACAT<br>GTATTACCAGTTGGTAATCGTAATAAACCAAACAAAAACCCTGTTTTATTGC<br>CTTTGAATTGGACTAGATTTCATTTAATGAAAGAATTCATGGCGATTTCAAA<br>ACCTTTCAACTACTTACCACCGGTCCAAGTTGGTGTAAACATTAACTATATC<br>CACCCACCACCTTTCATCCCACATTAATGCATCCCTCTCTCTCTATATATATG<br>TATGTATGGTGGTGGTTCTTCACCGTTACACCAGGTCTTTTAA

Table S2. Potential cis-acting elements in the pGhFDH promoter sequence using the PLACE and PlantCARE databases.

Program	Motif annotation	Motif seq	Position	Srand
PlantCARE	AAGAA-motif	GAAAGAA	-171	+
PLACE	ANAERO1CONSENSUS	AAACAAA	-345	+
PLACE	ANAERO1CONSENSUS	AAACAAA	-221	+
PLACE	ANAERO2CONSENSUS	AGCAGC	-530	-
PLACE	ANAERO2CONSENSUS	AGCAGC	-438	-
PlantCARE	ARE	AAACCA	-709	+
PlantCARE	ARE	AAACCA	-551	-
PlantCARE	ARE	AAACCA	-227	+
PLACE	ARR1AT	NGATT	-816	-
PLACE	ARR1AT	NGATT	-682	-
PLACE	ARR1AT	NGATT	-674	+
PLACE	ARR1AT	NGATT	-664	-

PLACE	ARR1AT	NGATT	-639	-
PLACE	ARR1AT	NGATT	-630	+
PLACE	ARR1AT	NGATT	-591	-
PLACE	ARR1AT	NGATT	-392	-
PLACE	ARR1AT	NGATT	-235	-
PLACE	ARR1AT	NGATT	-184	+
PLACE	ARR1AT	NGATT	-156	+
PlantCARE	ATCT-motif	AATCTAATCC	-190	-
PLACE	BIHD1OS	TGTCA	-794	+
PlantCARE	Box 4	ATTAAT	-73	-
PlantCARE	CAAT-BOX	CCAAT	-757	-
PlantCARE	CAAT-BOX	CAAAT	-375	-
PlantCARE	CAAT-BOX	CAAAT	-358	-
PlantCARE/PLACE	CAAT-BOX	CAAT	-673	-
PlantCARE/PLACE	CAAT-BOX	CAAT	-399	+
PlantCARE/PLACE	CAAT-BOX	CAAT	-394	+
PlantCARE/PLACE	CAAT-BOX	CAAT	-204	-
PlantCARE/PLACE	CAAT-BOX	CCAAT	-193	-
PLACE	CAAT-BOX	CAAT	-756	-
PLACE	CACTFTPPCA1	YACT	-699	-
PLACE	CACTFTPPCA1	YACT	-562	-
PLACE	CACTFTPPCA1	YACT	-523	+
PLACE	CACTFTPPCA1	YACT	-520	+
PLACE	CACTFTPPCA1	YACT	-502	-
PLACE	CACTFTPPCA1	YACT	-407	+
PLACE	CACTFTPPCA1	YACT	-352	-
PLACE	CACTFTPPCA1	YACT	-136	+
PLACE	CARGCW8GAT	CWWWWWWWWG	-389	+
PLACE	CTRMCAMV35S	TCTCTCTCT	-59	+
PLACE	DOFCOREZM	AAAG	-717	+
PLACE	DOFCOREZM	AAAG	-595	+
PLACE	DOFCOREZM	AAAG	-555	-
PLACE	DOFCOREZM	AAAG	-540	-
PLACE	DOFCOREZM	AAAG	-526	-
PLACE	DOFCOREZM	AAAG	-499	+
PLACE	DOFCOREZM	AAAG	-425	+
PLACE	DOFCOREZM	AAAG	-368	-
PLACE	DOFCOREZM	AAAG	-329	+

PLACE	DOFCOREZM	AAAG	-269	-
PLACE	DOFCOREZM	AAAG	-198	-
PLACE	DOFCOREZM	AAAG	-169	+
PLACE	DOFCOREZM	AAAG	-144	-
PLACE	DOFCOREZM	AAAG	-84	-
PLACE	DOFCOREZM	AAAG	-5	-
PLACE	DPBFCOREDCDC3	ACACNNG	-14	+
PLACE	EBOXBNNAPA	CANNTG	-440	+
PLACE	EBOXBNNAPA	CANNTG	-243	+
PLACE	EECCRCAH1	GANTTNC	-819	-
PLACE	EECCRCAH1	GANTTNC	-629	+
PLACE	ERELEE4	AWTTCAAA	-197	-
PLACE	ERELEE4	AWTTCAAA	-154	+
PLACE	EVENINGAT	AAAATATCT	-778	+
PLACE	GATABOX	GATA	-774	-
PLACE	GATABOX	GATA	-752	+
PLACE	GATABOX	GATA	-473	+
PLACE	GATABOX	GATA	-325	+
PLACE	GATABOX	GATA	-313	-
PLACE	GATABOX	GATA	-264	-
PLACE	GATABOX	GATA	-98	-
PlantCARE	GATA-motif	AAGGATAAG G	-329	+
PLACE	GT1CONSENSUS	GRWAAW	-819	+
PLACE	GT1CONSENSUS	GRWAAW	-790	-
PLACE	GT1CONSENSUS	GRWAAW	-782	+
PLACE	GT1CONSENSUS	GRWAAW	-714	+
PLACE	GT1CONSENSUS	GRWAAW	-685	+
PLACE	GT1CONSENSUS	GRWAAW	-628	-
PLACE	GT1CONSENSUS	GRWAAW	-292	+
PLACE	GT1CONSENSUS	GRWAAW	-248	-
PLACE	GT1CONSENSUS	GRWAAW	-238	+
PLACE	GT1CORE	GGTTAA	-723	-
PlantCARE	GT1-motif	GGTTAA	-724	-
PLACE	GTGANTG10	GTGA	-298	-
PLACE	GTGANTG10	GTGA	-262	-
PLACE	GTGANTG10	GTGA	-22	-
PLACE	INRNTPSADB	YTCANTYY	-321	-
PlantCARE	MYB	TAACCA	-723	+
PlantCARE	Myb	CAACTG	-244	-
PLACE	MYB1AT	WAACCA	-722	+
PLACE	MYB1AT	WAACCA	-708	+
PLACE	MYB1AT	WAACCA	-550	-
PLACE	MYB1AT	WAACCA	-226	+

PlantCARE/PLACE	MYB1AT	WAACCA	-491	-
PLACE	MYBCORE	CNGTTR	-678	-
PLACE	MYBCORE	CNGTTR	-243	+
PLACE	MYBCORE	CNGTTR	-19	+
PLACE	MYBPLANT	MACCWAMC	-225	+
PLACE	MYBST1	GGATA	-753	+
PLACE	MYBST1	GGATA	-326	+
PLACE	MYBST1	GGATA	-98	-
PLACE	MYCCONSENSUSAT	CANNTG	-564	-
PLACE	MYCCONSENSUSAT	CANNTG	-243	+
PLACE	NTBBF1ARROLB	ACTTTA	-270	+
PLACE	P1BS	GNATATNC	-734	+
PLACE	P1BS	GNATATNC	-316	+
PlantCARE	P-box	CCTTTTG	-428	-
PLACE	POLASIG1	AATAAA	-759	-
PLACE	POLASIG1	AATAAA	-511	-
PLACE	POLASIG1	AATAAA	-377	-
PLACE	POLASIG1	AATAAA	-229	+
PLACE	POLASIG1	AATAAA	-206	-
PLACE	POLASIG3	AATAAT	-360	-
PLACE	POLLEN1LELAT52	AGAAA	-783	+
PLACE	POLLEN1LELAT52	AGAAA	-715	+
PLACE	POLLEN1LELAT52	AGAAA	-293	+
PLACE	PRECONSCRHSP70A	SCGAYNRNNNNNNNNNNNNNNNHD	-675	+
PLACE	PYRIMIDINEBOXOSRAMY1A	CCTTTT	-426	-
PLACE	RAV1AAT	CAACA	-810	+
PLACE	RAV1AAT	CAACA	-609	+
PLACE	RAV1AAT	CAACA	-604	-
PLACE	RBCSCONSENSUS	AATCCAA	-682	+
PLACE	REALPHALGLHCB21	AACCAA	-721	+
PLACE	REALPHALGLHCB21	AACCAA	-599	+
PLACE	ROOTMOTIFTAPOX1	ATATT	-776	-
PLACE	ROOTMOTIFTAPOX1	ATATT	-315	-
PLACE	RYREPEATBNNAPA	CATGCA	-704	+
PLACE	SEF3MOTIFGM	AACCCA	-697	-
PLACE	SEF3MOTIFGM	AACCCA	-613	+
PLACE	SEF3MOTIFGM	AACCCA	-381	-
PLACE	SEF4MOTIFGM7S	RTTTTTR	-348	-
PLACE	SEF4MOTIFGM7S	RTTTTTR	-218	-
PLACE	SORLIP1AT	GCCAC	-737	-

PLACE	SV40COREENHAN	GTGGWWHG	-134	-
PLACE	TAAAGSTKST1	TAAAG	-540	-
PLACE	TAAAGSTKST1	TAAAG	-526	-
PLACE	TAAAGSTKST1	TAAAG	-500	+
PlantCARE	TATA-BOX	TATA	-732	+
PlantCARE	TATA-BOX	TATA	-267	-
PlantCARE	TATA-BOX	TATA	-101	-
PlantCARE	TATA-BOX	TATATA	-52	-
PlantCARE	TATA-BOX	TATATA	-48	-
PlantCARE/PLACE	TATA-BOX	ccTATAAAaa	-482	-
PlantCARE/PALCE	TATA-BOX	TATTAAT	-692	+
PLACE	TATA-BOX	TTATTT	-510	+
PLACE	TATA-BOX	TTATTT	-376	+
PLACE	TATA-BOX	TTATTT	-359	+
PLACE	TATCCAOSAMY	TATCCA	-754	-
PLACE	TATCCAOSAMY	TATCCA	-98	+
PlantCARE	Unnamed__4	CTCC	-423	-
PlantCARE	Unnamed__4	CTCC	-416	+
PlantCARE	Unnamed__4	CTCC	-406	+
PlantCARE	Unnamed__4	CTCC	-402	+
PlantCARE	WRE3	CCACCT	-89	+
PLACE	WRKY71OS	TGAC	-793	-
PLACE	WUSATAg	TTAATGG	-690	+
PlantCARE		motif_sequence	-624	+
PlantCARE		motif_sequence	-471	+
PlantCARE		motif_sequence	-365	-

Table S3. Primer Sequences.

Primer name	Primer sequence（5′-3′）	Note
BP-pGhFDH-F	GGGGACAAGTTTGTACAAAAAAGCAGGCTGCCTTGC TCGGCTGTCATTTTCG	Expression vector construction
BP-pGhFDH-R	GGGGACCACTTTGTACAAGAAAGCTGGGTGATTAAA AGACCTGGTGTAACGGTG	
GhUB7-F	GAAGGCATTCCACCTGACCAAC	qRT-PCR
GhUB7-R	CTTGACCTTCTTCTTCTTGTGCTTG	
GhFDH-qRT-F	TCGATGAAGCGAGTCTTGAA	
GhFDH-qRT-R	CCGAGGTTGTAGCTCAAAAT	
M13-F	CGCCAGGGTTTTCCCAGTCACGAC	E. coli positive test
M13-R	CACACAGGAAACAGCTATGAC	
NPTII-F	GTCATACCACTTGTCCGCCCT	Agrobacterium tumefacienspositive test
NPTII-R	CAGCCGGTATAAAGGGACCACC	
GUS-F	TGTAACCACGCGTCTGTTGACTG	GUS genetic testing
GUS-R	AATACTCCACATCACCACGC	

Table S4. GUS activity in different tissues and developmental periods.

	Line	Tissue	1	2	3	Average value
Arabidopsis thaliana	*CaMV35S ::GUS*	Root	7253.5	7116.13	7319.64	7229.756667
		Stalk	9202.3	9134.11	9333.96	9223.456667
		Leaf	6531.07	6479.23	6490.3	6500.2
		Inflorescence	5799.51	5988.32	5978.92	5922.25
		Pod	3816.11	3752.98	3789.25	3786.113333
	pGhFDH: :GUS-1	Root	6800.3	6671.3	6417.1	6629.566667
		Stalk	9216.25	9365.21	9319.88	9300.446667
		Leaf	6319.12	6264.25	6119.89	6234.42
		Inflorescence	8481.4	8215.3	7799.97	8165.556667
		Pod	1998.51	2141.87	1923.64	2021.34
	pGhFDH: :GUS-2	Root	8520.16	8560.89	8546.66	8542.57
		Stalk	7762.28	7796.35	7782.16	7780.263333
		Leaf	5919.91	5917.08	5841.64	5892.876667
		Inflorescence	6835.1	6834.81	6820.66	6830.19
		Pod	2017.09	2106.38	2019.82	2047.763333
Cotton	*CaMV35S ::GUS*	Leaf	6532.01	6621.12	6561.14	6571.423333
		Stalk	13854.22	13776.02	13655.79	13762.01
		Bud	16901.63	16814.47	16892.96	16869.68667
		Petal	9570.81	9568.31	9544.23	9561.116667
		Anther	28031.29	28315.56	28115.61	28154.15333
		Shoot tip	6281.94	6221.33	6257.66	6253.643333

	0 DPA	4682.33	4671.59	4661.66	4671.86
	5 DPA	3259.36	3126.01	3216.01	3200.46
	10 DPA	1672.31	1668.96	1669.25	1670.173333
	20 DPA	3603.33	3608.65	3587.14	3599.706667
	30 DPA	5846.23	5755.55	5799.21	5800.33
	Mature fiber	900.00	887.36	881.31	889.5566667
	Leaf	8537.36	8716.59	8605.69	8619.88
	Stalk	5406.28	5395.79	5398.12	5400.063333
	Bud	15635.57	15580.29	15643.59	15619.81667
	Petal	14931.20	14952.33	14907.02	14930.18333
	Anther	30321.5	30305.59	30304.25	30310.44667
pGhFDH:	Shoot tip	4635.55	4636.17	4622.26	4631.326667
:GUS-1	0 DPA	7956.33	7954.12	7946.76	7952.403333
	5 DPA	4779.56	4784.26	4632.93	4732.25
	10 DPA	12615.26	12510.36	12465.10	12530.24
	20 DPA	32150.69	31962.33	31887.84	32000.28667
	30 DPA	17532.59	17398.46	17462.83	17464.62667
	Mature fiber	1231.54	1233.12	1136.56	1200.406667
	Leaf	7547.66	7616.53	7695.65	7619.946667
	Stalk	6854.32	6802.16	6745.01	6800.496667
	Bud	13967.2	13864.4	13847.6	13893.06667
	Petal	16760.81	16763.56	16771.33	16765.23333
	Anther	31269.55	31267.84	31236.86	31258.08333
pGhFDH:	Shoot tip	3987.41	4031.87	3866.37	3961.883333
:GUS-2	0 DPA	8406.25	8238.36	8326.22	8323.61
	5 DPA	3935.35	3892.01	3842.54	3889.966667
	10 DPA	10603.52	10514.16	10352.97	10490.21667
	20 DPA	36213.05	36138.79	36009.2	36120.34667
	30 DPA	18149.31	18138.08	18316.2	18201.19667
	Mature fiber	1318.26	1328.36	1305.79	1317.47
